# A Guild‐Based Ecological Assessment: Integrating Multi‐Model Approaches to Reveal Key Environmental Associations for Waterbirds in Nansi Lake, China

**DOI:** 10.1002/ece3.73694

**Published:** 2026-05-21

**Authors:** Sai Jiang, Yuewei Yang, Fengfei Sun, Xingjia Zheng, Wei He, Meizhen Tang, Junfeng Chen, Shiqiang Ma, Gang Li

**Affiliations:** ^1^ School of Life Science Qufu Normal University Qufu Shandong China

**Keywords:** machine learning species random effects, Nansi Lake, nutrient enrichment, waterbird guilds

## Abstract

Wetlands are increasingly threatened by biodiversity loss and functional degradation. Waterbirds guilds, which reflect functional rather than taxonomic organization, provide a potentially informative yet underexplored framework for wetland assessment. In this study, we employed a multi‐model approach in Nansi Lake, northern China. Environmental gradients were derived via principal component analysis (PCA), and relationships with waterbird guild densities were examined using Tweedie generalized linear mixed models (GLMM), with species included as a random effect to account for baseline density differences. Parallel random forest (RF) and LASSO regressions were applied to explore robust associations. GLMMs revealed that guild densities were consistently associated with the first two PCA gradients: PC1 (representing phytoplankton–zooplankton contrasts) and PC2 (high dissolved oxygen, low nitrogen). Notably, piscivorous birds showed a positive response to PC2 compared to other feeding guilds. Species identity explained a large proportion of density variation, confirming that controlling for interspecific differences was essential for detecting these environmental signals. Nutrient‐related variables and sediment conditions exhibited negative associations with waterbird guild metrics across models, whereas fish diversity and periphyton‐related variables exerted positive influences. The convergence of association patterns between linear mixed models and nonlinear machine learning methods increased our confidence, despite limited sample size. These findings suggest that guild‐level patterns reflect the combined influence of abiotic conditions and biotic resource availability. Waterbird guild metrics offer useful, scalable indicators of wetland ecological condition, with potential applications in monitoring, assessment and conservation strategies.

## Graphical abstract

In Nansi Lake, waterbird guild densities were consistently linked to environmental gradients, with negative responses to nutrients and sediment variables, but positive responses to fish diversity and periphyton. Piscivores specifically favored high dissolved oxygen and low nitrogen conditions. These guild‐level patterns offer practical, scalable indicators for wetland monitoring and assessment.

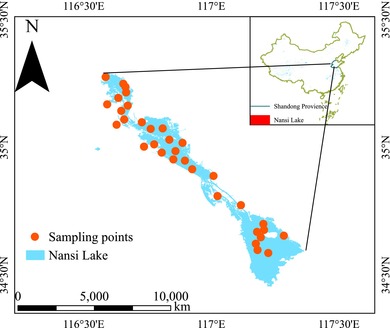

## 1 Introduction

Waterbirds respond rapidly to changes in resource availability and habitat conditions through their high mobility and aggregative behavior, making them sensitive indicators of wetland ecosystem dynamics (Cumming et al. 2012). Owing to their diversity, abundance, and sensitivity to aquatic environments, waterbirds are widely used to reflect changes in water quality, trophic status, vegetation dynamics, and changes in prey abundance (Aarif et al. 2025; Batanero et al. 2017; Boros et al. 2023; Wen et al. 2016). Rather than responding uniformly, however, waterbirds often exhibit heterogeneous ecological responses that are structured by their functional roles within communities.

The guild concept describes groups of species that use similar resources in similar ways and provides a functional framework for understanding avian community organization (Grinnell 1917; Root 1967). Classifying birds into guilds based on foraging strategies, diet, and habitat use provides a different lens for assessing community‐level responses to environmental change compared with analyses focused on individual species. Species within the same guild tend to exhibit similar ecological responses, whereas contrasting responses are often observed among different guilds when environmental conditions change (Xu et al. 2024). Consequently, guild‐based approaches are increasingly recognized as robust tools for linking avian community patterns to ecosystem processes.

In aquatic ecosystems, the relationships between waterbird guild abundance and community composition and their environmental variables are often complex and nonlinear. Nutrient enrichment, water clarity and changes in aquatic communities are frequently interrelated and tend to co‐occur with changes in food availability and habitat structure, which are commonly associated with shifts in guild composition. For example, eutrophication may enhance primary productivity under certain conditions but can also degrade habitat quality and reduce biodiversity when nutrient loading exceeds ecological thresholds (Cardinale et al. 2009; Tittensor et al. 2010), Waterbird guilds are indirectly regulated by such processes through trophic interactions with fish, invertebrates and aquatic vegetation (Aarif et al. 2025; Guo et al. 2019; Xie et al. 2020). Identifying the key environmental variables associated with these patterns therefore requires analytical methods capable of addressing multicollinearity and nonlinear relationships.

Nansi Lake, the largest freshwater lake in northern China, is a shallow marsh‐type lake of high ecological importance and a key wetland node along the East Asian–Australasian Flyway. Its extensive shallow waters and warm‐temperate monsoon climate support diverse aquatic communities and provide critical habitat for migratory and resident waterbirds (Zheng et al. 2023). However, despite long‐term pollution control efforts, Nansi Lake has experienced increasing nutrient enrichment in recent decades (J. Liu et al. 2024). Water quality monitoring indicates a shift from mesotrophic to mildly eutrophic conditions, driven primarily by nitrogen inputs and chemical oxygen demand associated with surface runoff and anthropogenic activities (Bing et al. 2001; Li et al. 2025; Liu and Zheng 2002). Previous studies in Nansi Lake have largely focused on water quality assessment and descriptive surveys of waterbird diversity and community composition (Li et al. 2017; Liu et al. 2023). Although waterbird foraging guilds are widely recognized as integrative indicators of wetland ecosystem condition, guild‐based approaches have not yet been incorporated into ecological assessments of Nansi Lake. In particular, the relationships between waterbird guild structure, physicochemical properties, and aquatic community characteristics remain poorly understood. This lack of guild‐level, process‐oriented analysis limits our ability to link waterbird assemblages to underlying ecological drivers and constrains the application of functional indicators for wetland monitoring and management. Addressing this knowledge gap is essential for improving ecosystem‐based assessment frameworks in nutrient‐enriched shallow lakes such as Nansi Lake.

To better understand how environmental variation shapes waterbird guild structure, this study examines the relationships between waterbird guilds and a range of water quality parameters and aquatic community characteristics in the Nansi Lake riparian zone. Specifically, we address the following questions: (1) How are waterbird guilds composed and classified in the Nansi Lake riparian zone? (2) Which environmental and aquatic community factors are most strongly associated with different waterbird guilds? (3) Do different analytical approaches identify similar patterns of associations between waterbird guilds and environmental factors? To address these questions, we applied a multi‐model framework combining Principal Component Analysis (PCA) with Tweedie Generalized Linear Mixed Models (GLMM), complemented by random forest (RF) (Cutler et al. 2007) and LASSO (Ranstam and Cook 2018) regression. In the GLMMs, species was included as a random intercept to control for inherent baseline density differences between gregarious and territorial taxa. These methods differ in their underlying assumptions, ranging from linear responses to complex interactions, and provide a robust framework for exploring guild–environment relationships and improving the ecological interpretation of guild‐based indicators.

## 2 Materials and Methods

### 2.1 Sample Collection

#### 2.1.1 Data Acquisition and Guild Classification

**FIGURE 1:**
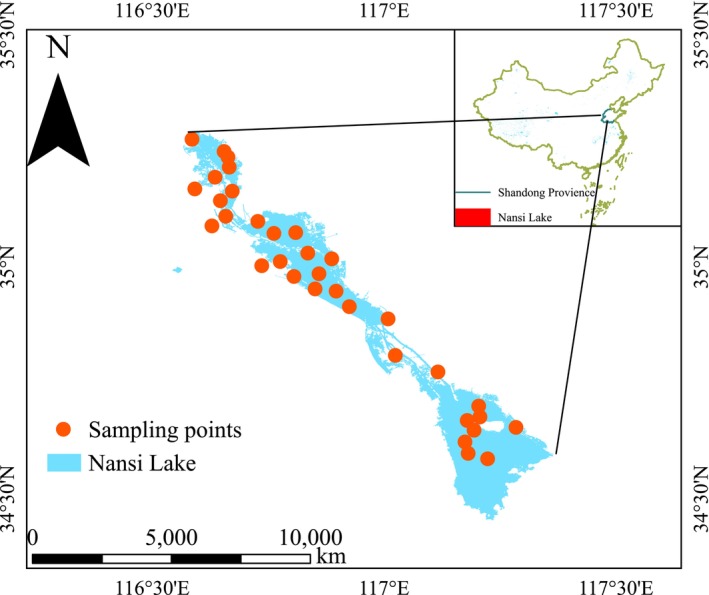
Geographic location of Nansi Lake in northern China (inset map) and spatial distribution of sampling sites used for waterbird surveys and aquatic environmental measurements. Some sampling sites were located along the lakeshore, near river inflows, which may appear relatively distant from the shore in the figure. Other sites were situated in the open water areas of the lake.

From June to August 2022, the research team conducted waterbird surveys along the lakeshore and in open water areas of Nansi Lake, using binoculars (8 × 42, 10 × 42) and spotting scopes. Surveys were conducted during peak bird activity hours (06:30–10:30 and 15:00–18:00) on clear, windless days. The observation area covered a circular region with the plot as the center and a radius of 300 m. Observers simultaneously recorded the species and number of all individuals remaining within the sampling plot and those flying out of it (individuals flying into the plot were not counted). In open water plots, observers conducted visual counts from fixed points for sufficient time to ensure that most individuals were recorded. In plots with potential visual obstruction, such as shoreline shrubs, observers combined visual observation with acoustic detection and approached individuals when necessary to confirm species identity and numbers. Each survey lasted approximately 15 min per site. The surveys were conducted on foot and by vehicle. As some sampling sites were located on the open water of the lake, we accessed the corresponding geographic coordinates by boat. A total of 33 sampling sites were randomly selected for the collection of data on waterbirds, physicochemical water quality, and aquatic biotic communities (Figure 1), with a minimum distance of 500 m between sites to reduce spatial overlap and ensure independent sampling.

Species classification and nomenclature followed Gill et al. (2022). Waterbird guilds were classified into three sets of guilds according to three classification approaches. The first approach classified waterbird guilds using the importer‐exporter (IMEX) and net‐exporter (EX) guild system proposed by Boros (2021), which focuses on the location of feeding habitats and the quantitative role of waterbirds in the nutrient and energy flow for inland aquatic ecosystems. The classification represents an integrated approach that combines taxonomic attributes, trophic positions, foraging behavior, and patterns of daily habitat use, drawing on the most relevant previous studies and reference datasets related to waterbird ecology and nutrient cycling. Second, waterbirds were classified into three feeding guilds based on dietary preferences: carnivorous (CAR), omnivorous (OMN), and piscivorous (PIS). Third, waterbirds were classified by ecological type into Waders (W) and Waterfowl (WF). Waders were represented by species from Pelecaniformes and Charadriiformes, excluding gulls. Waterfowl were represented by species from Suliformes, Gruiformes, Anseriformes, Podicipediformes, and gulls. Bird density (ind/km^2^) was calculated based on the total number of individuals recorded within a 300 m radius during a single survey at each site. Individuals were assigned to guilds following the three classification schemes described above. And the total density of each guild was used as the response variable in subsequent analyses.

#### 2.1.2 Environmental Variables and Aquatic Community Index

All 33 sampling sites were surveyed within the same year. At each site, waterbird surveys were conducted concurrently with the collection of physicochemical water parameters and aquatic community. Water quality parameters were recorded, including ammonia nitrogen (AN), chlorophyll a (Chla), total organic carbon (TOC), transparency (T), conductivity (C), sediment organic matter (SOM), dissolved oxygen (DO), total nitrogen (TN), water temperature (WT), pH, and total phosphorus (TP).

Aquatic community indices were collected and quantified as follows: Zoobenthos diversity (Zoob_Div), biomass (Zoob_Bio), and density (Zoob_Den); Zooplankton diversity (Zoop_Div), biomass (Zoop_Bio), and density (Zoop_Den); Phytoplankton diversity (Phyt_Div), biomass (Phyt_Bio), and density (Phyt_Den); Periphyton (attached algae) diversity (Att_Div) and density (Att_Den); and Fish diversity (Fish_Div).

### 2.2 Research Methods

#### 2.2.1 Statistical Analyses

Normality of waterbird density, aquatic indices and water quality parameters was assessed using the Shapiro–Wilk and Kolmogorov–Smirnov tests (with Lilliefors correction). Variables that were not normally distributed were analyzed using nonparametric Wilcoxon rank‐sum tests. Density values were log (*x* + 1)‐transformed prior to analysis to reduce skewness and accommodate zero values.

Spearman correlation analysis was conducted to evaluate relationships among environmental variables (physicochemical properties, plankton, periphyton, and zoobenthos) (Table A1). Visualizations were created using the Chiplot online platform (https://www.chiplot.online/). All statistical analyses were conducted in R (version 4.4.2) (R Development Core Team 2018).

#### 2.2.2 Random Forest

Developed by Breiman (2001), RF is a machine learning method for classification and regression. RF builds an ensemble of Classification and Regression Trees (CART) to form a nonlinear predictive model with high efficiency and accuracy. The method does not assume independence among predictors or specific data distributions, making it robust to noise and applicable to both large and relatively small datasets (Breiman 2001; Cutler et al. 2007). Each tree is constructed using multiple independent bootstrap samples. Random feature selection is applied at each node split (Liaw and Wiener 2002). Though random feature selection may slightly increase model bias, variance reduction through ensemble averaging typically improves model performance. Variable importance is assessed using two metrics: %IncMSE, representing the increase in prediction error on out‐of‐bag (OOB) samples when a variable is permuted, with higher values indicating greater importance; and IncNodePurity, representing the total reduction in node impurity due to a variable.

In this study, %IncMSE was used as the importance criterion. For each response variable, model hyperparameters were optimized through a grid search over mtry (*p*/3, where *p* is the number of predictors), maximum tree depth (1–12), minimum sampled to split a node (10–20), and minimum samples per leaf (10–16). RF was implemented in R with the “randomForest” package (Breiman 2001).

#### 2.2.3 LASSO Regression

Least absolute shrinkage and selection operator (LASSO) regression was used to simultaneously perform variable selection and regularization, thereby improving model generalizability and preventing overfitting. An L1 penalty was applied to the regression coefficients, causing some to be shrunk to zero and producing sparse models.

The objective function is minimized:

$$\min_{\beta }\left\{\underset{\text{Mean squared error}\;\left(\mathrm{MSE}\right)}{\underbrace{\frac{1}{2N}\sum_{i=1}^{N}{\left(y_{i}-\beta_{0}-\sum_{j=1}^{p}\beta_{j}x_{\mathrm{ij}}\right)}^{2}}}+\underset{L1\;\text{penalty term}}{\underbrace{\lambda \sum_{j=1}^{p}\left|\beta_{j}\right|}}\right\}$$

where: *N* is the number of samples, p is the number of predictors, $y_{i}$ is the observed response, $x_{\mathrm{ij}}$ is the value of feature j for observation i, $\beta_{0}$ is the intercept, $\beta_{j}$ is the coefficient for feature j, λ is the regularization parameter. Too large a *λ* leads to underfitting (all $\beta_{j}$ = 0), while too small a λ approximates OLS, risking overfitting.

The procedure was conducted as follows: First, all predictor variables were standardized using *z*‐score normalization before model fitting. Specifically, for each variable, the mean of the training set was subtracted and the result divided by the standard deviation of the training set. The same parameters (mean and standard deviation) were then applied to the test set. This ensures that variables with different units or ranges contribute equally to the LASSO model and allows meaningful comparison of coefficient magnitudes during feature selection. Second, the dataset was split into train set (70%) and test set (30%). Third, the optimal λ was selected via 5‐fold cross‐validation using the cv.glmnet function in R. Then, the model was fitted using the optimal *λ*. Fourth, predicitive accuracy was assessed using *R* ^2^. Finally, feature selection was performed by retaining coefficients that were nonzero with their magnitudes reflecting variable importance. LASSO regression was implemented using the “glmnet” package in R v 4.4.2 (Friedman et al. 2010).

#### 2.2.4 PCA and Tweedie GLMM

We conducted a PCA on environmental and biotic variables. All variables were standardized (*z*‐score) prior to analysis. PCA was performed using the prcomp function, and the first three principal components (PC1–PC3) were extracted. The variance explained by each component and the cumulative variance were calculated. Variable loadings were used to identify major driving factors, with |loading| > 0.2 as the threshold for selecting important contributors, facilitating ecological interpretation of the principal components. Site scores from PCA were subsequently used for regression analyses.

Three guild classification schemes were examined. In the first scheme, birds were grouped as W and WF (guild_1); in the second, as CAR, PIS, and OMN (guild_2); and in the third, as IMEX and EX (guild_3). For each scheme, a main‐effects model (PC1 + PC2 + PC3 + guild) and an interaction model [(PC1 + PC2 + PC3) × guild] were fitted. To prevent the analysis from being dominated by a few gregarious species, all models included species as a random intercept (1|species) (Schall 1991; Warton et al. 2015). This specification accounts for intrinsic differences in baseline density among species, thereby preventing abundant species from unduly influencing the estimated environmental effects and ensuring that fixed‐effect estimated are conditional on species composition (Bolker et al. 2009; Jamil et al. 2013). Models were fitted assuming a Tweedie distribution with a log link using the glmmTMB package. Model selection was based on AIC and marginal/conditional *R* ^2^, with the latter computed using the MuMIn package. The variance of the species‐level random intercept was inspected to evaluate the magnitude of inter specific variation relative to the fixed effects. All analyses in this study were performed in R (version 4.4.2) (R Development Core Team 2018).

## 3 Results

### 3.1 Composition of Waterbird Guilds

A total of 17 waterbird species were identified, belonging to the following orders: Charadriiformes (*n* = 2), Pelecaniformes (*n* = 9), Gruiformes (*n* = 2), Suliformes (*n* = 1), Anseriformes (*n* = 1), Podicipediformes (*n* = 2). The detailed species composition was presented in the Supporting Information (Table A1).

**FIGURE 2:**
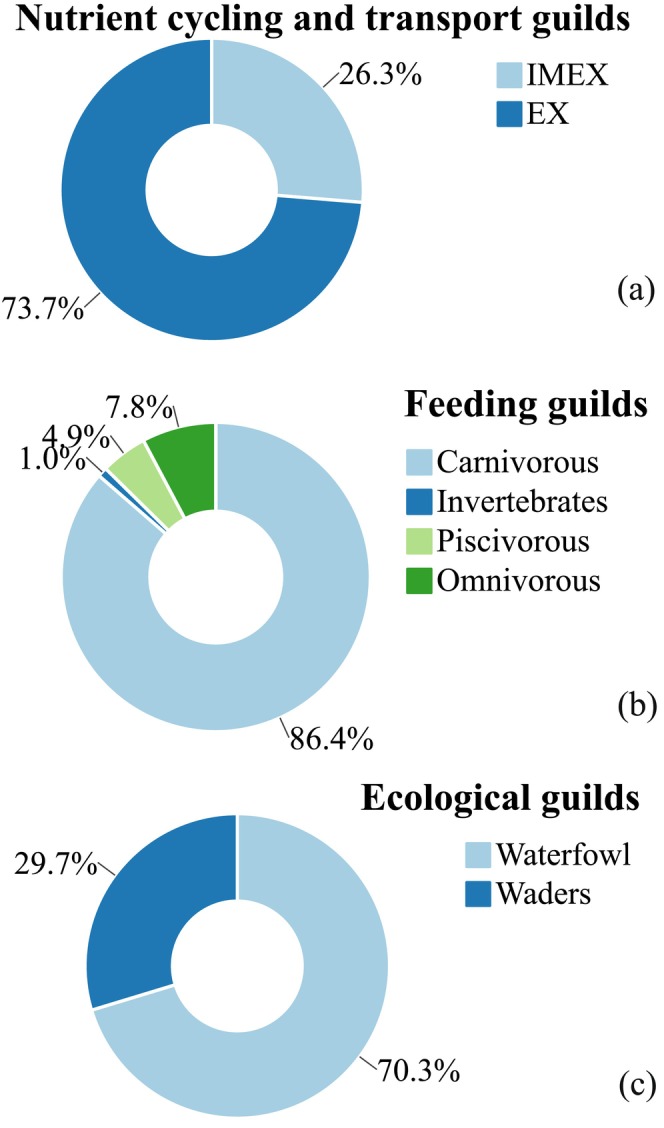
Percentage composition of waterbird guilds. (a) Nutrient cycling and transport guilds were grouped into import‐exporter guild (IMEX) and net‐exporter guild (EX). (b) Feeding guilds include carnivorous, piscivorous, and omnivorous. (c) Ecological guilds were divided into waterfowl and waders.

**FIGURE 3:**
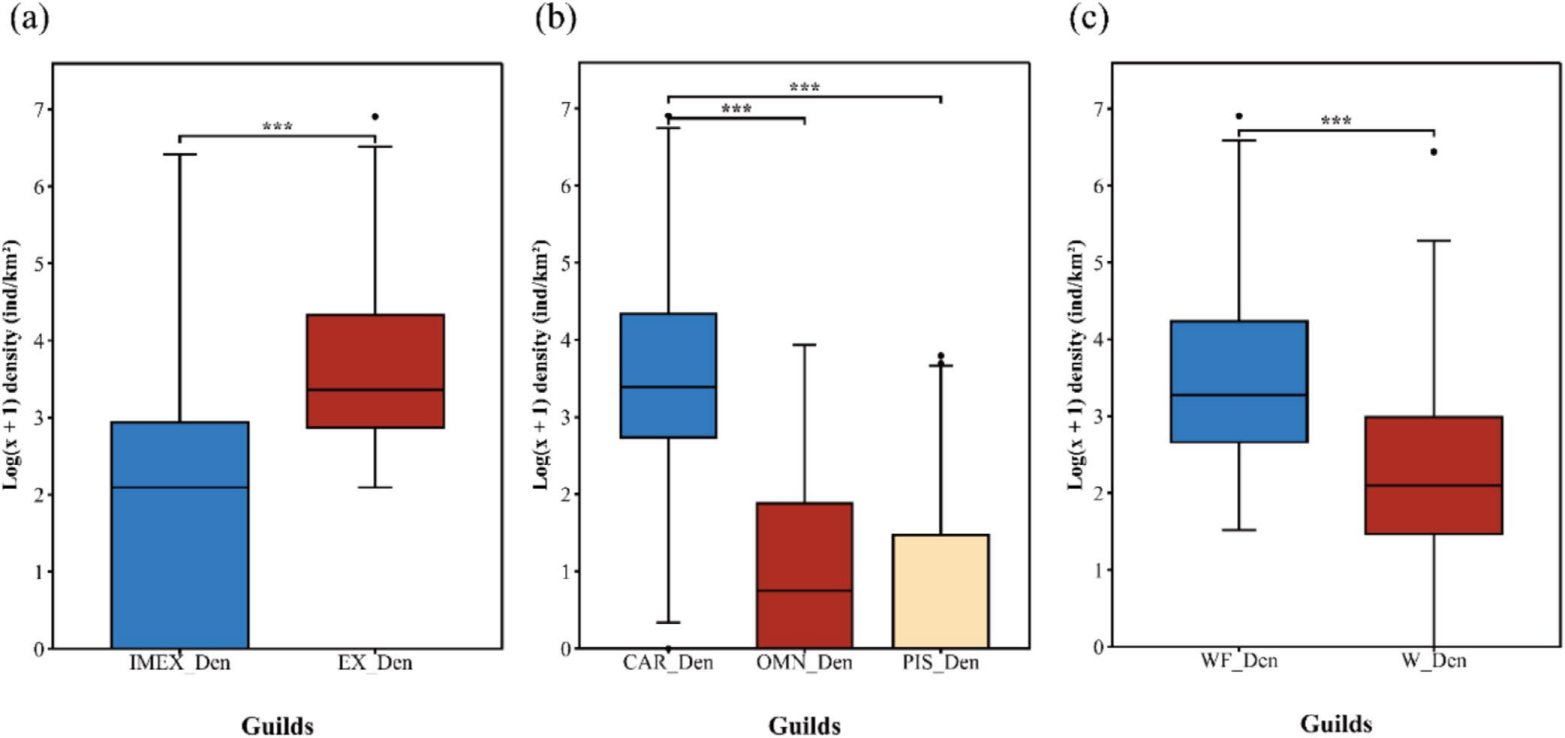
Distribution of log (*x* + 1)‐transformed waterbird density among guilds. (a) Nutrient cycling and transport guilds. (b) Feeding guilds. (c) Ecological guilds. Significance markers indicated pairwise differences across guilds, assessed using Wilcoxon rank‐sum tests with Bonferroni correction. Significance was indicated as follows: ***p ≤ 0.001.

Guild densities were found to follow non‐normal distributions, necessitating the use of Wilcoxon rank‐sum tests (Table A3). Among feeding guilds, carnivorous (CAR) was dominant in density. For ecological guilds, waders (W) showed higher density than waterfowl (WF) (Figure 2). Within nutrient cycling and transport guild, net‐exporter (EX) showed significantly higher density than importer‐exporter (IMEX) (*p* < 0.001; Figure 3).

### 3.2 Characteristics of Environmental Factors and Aquatic Biota

Descriptive statistics for environmental variables were presented in Table 1. The lake was generally shallow (mean depth 6.0 m) with moderate conductivity. Total nitrogen (TN) and total phosphorus (TP) concentrations varied among sites, while Chla ranged from oligotrophic to hypereutrophic levels. Zooplankton and benthic animal densities showed large variation, with phytoplankton and benthic algae generally dominating biomass. SOM also varied widely.

**TABLE 1:** Definitions and descriptive statistics of selected environmental variables and aquatic biotic community explanatory variables.

Variables	Mean (range)	Description
AN (mg/L)	1.01 (0.3–2.33)	Ammonia nitrogen
TP (mg/L)	0.19 (0.06–0.38)	Total phosphorus
TN (mg/L)	2.52 (0.7–4.29)	Total nitrogen
Chla (μg/L)	29.42 (5–155)	Density of Chlorophyll a
TOC (mg/L)	4.94 (3.5–7)	Total organic carbon
DO (mg/L)	5.87 (4.6–6.8)	Dissolved oxygen
WT (°C)	26.58 (24.1–27.9)	Water temperature
pH	7.56 (7.2–8.4)	
T (cm)	42.27 (0–73)	Transparency
C (μS/cm)	931.3 (478–1459)	Conductivity
SOM (g/kg)	66.39 (4.56–213.51)	Organic matter in river sediment
Zoob_Den (ind/m^2^)	1111.39 (8–6592)	Individual density of zoobenthos
Zoob_Bio (g/m^2^)	149.56 (0.21–1038.36)	Biomass density of zoobenthos
Zoob_Div	1.78 (0–3.16)	Species diversity of zoobenthos
Fish_Div	2.42 (1.3–3.29)	Species diversity of fish
Zoop_Den (ind/L)	141.89 (6.8–684)	Individual density of zooplankton
Zoop_Bio (mg/L)	0.87 (0.04–4.04)	Biomass density of zooplankton
Zoop_Div	2.98 (1.3–3.91)	Species diversity of zooplankton
Phyt_Den (10^4^ ind/L)	1116.28 (168.75–3372.9)	Individual density of phytoplankton
Phyt_Bio (mg/L)	13.2 (2.6–38.23)	Biomass density of phytoplankton
Phyt_Div	4.57 (3.99–5.07)	Species diversity of phytoplankton
Att_Den (10^4^ cells/cm^2^)	386 (10.99–1256.6)	Individual density of periphyton
Att_Div	3.5 (1.68–4.57)	Species diversity of periphyton

Spearman correlation analyses revealed several significant relationships (Figure S4). TP and TN were frequently correlated with aquatic biotic variables, while other parameters (e.g., DO, WT, Chla) showed guild‐specific associations.

### 3.3 Relationships Between Guilds and Environmental/Aquatic Factors

#### 3.3.1 Random Forest

OOB error stabilized once the number of trees (ntree) exceeded 300 (Figure S1). The lowest OOB errors and optimal parameters for each modeled guild were presented in Figure S1. In this study, many environmental variables have %IncMSE values above zero. These values indicate that these variables are related to waterbird guild metrics at different levels. The sample size in this study is limited. To avoid overinterpreting weak effects, this study focuses only on the top five variables ranked by importance for ecological interpretation.

**FIGURE 4:**
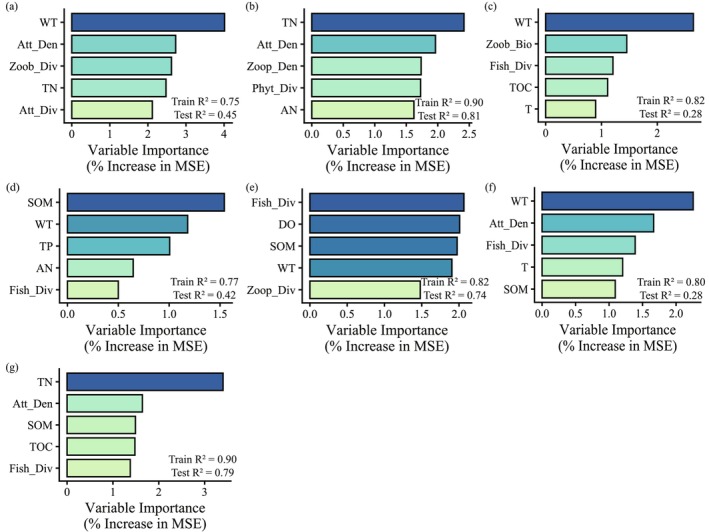
Top 5 variables ranked by %IncMSE importance in random forest models for each guild: (a) Import‑exporter, (b) Net‑exporter, (c) Omnivorous, (d) Piscivorous, (e) Carnivorous, (f) Waders, (g) Waterfowl. Complete importance rankings are shown in Figure S5.

RF analyses indicated that several environmental variables were consistently related to waterbird guild metrics (Figure 4). EX and WF achieved the highest model performance (Test *R* ^2^ = 0.81 and 0.79), indicating that the selected environmental and aquatic predictors effectively captured the key drivers of these guilds. While, OMN and W performed poorly (Test *R* ^2^ = 0.28 for both), suggesting that their distributions may be influenced by unmeasured variables or complex ecological interactions beyond the current predictors.

#### 3.3.2 Lasso

**FIGURE 5:**
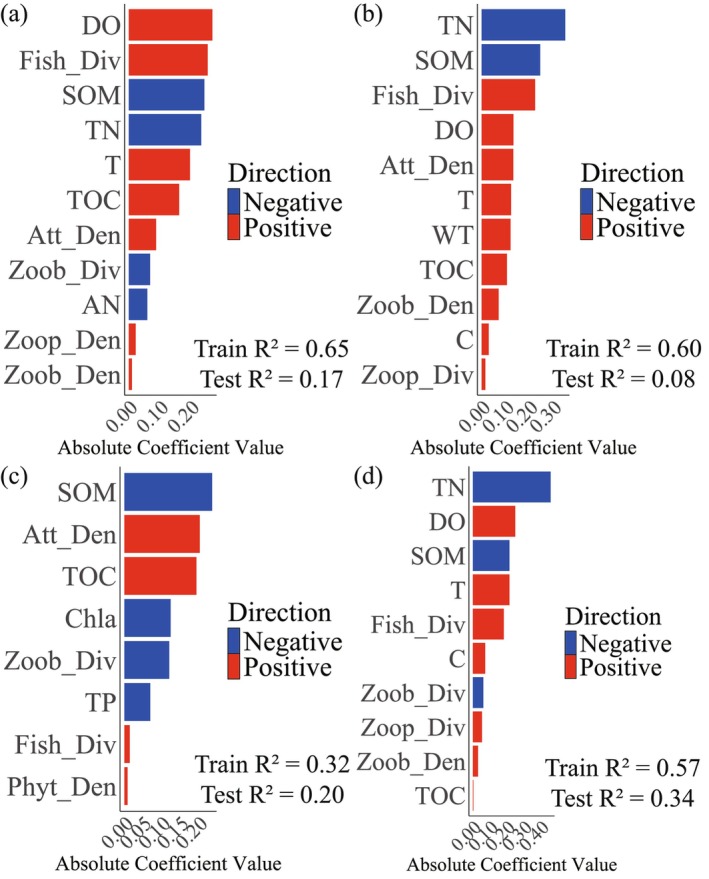
Predictor variable importance for each waterbird guild, ranked by absolute spearman correlation values. Positive and negative associations were distinguished by color coding. (a) Net‐exporter guild, (b) Carnivorous, (c) Waders, (d) Waterfowl. No effective models were obtained for the Import‐exporter guild, Piscivorous, and Omnivorous guilds.

The optimal regularization parameter (*λ*) for each guild model was determined via cross‐validation (Figure S2), and the coefficient paths were visualized in Figure S3. Feature selection was automatically performed by shrinking the coefficients of irrelevant predictors to zero, thereby retaining only informative variables. Variable importance was ranked based on the absolute Spearman correlation values between each predictor and the response variables, and the direction of influence was also assessed (Figure 5).

LASSO identified a smaller set of predictors through coefficient shrinkage (Figure 5). TN and SOM generally had negative associations with guild densities, while Fish_Div and DO were positive. EX and CAR models had the highest explanatory power in training data (*R* ^2^ = 0.65 and 0.60), but test *R* ^2^ values were lower, indicating some overfitting. Models for IMEX, PIS, and OMN could not be reliably established (Figure S3).

#### 3.3.3 PCA of Environmental and Biotic Variables

PCA was conducted on standardized environmental and biotic variables. The first three principal components (PCs) explained 44.8% of the total variance, with PC1 accounting for 20.7%, PC2 for 12.3%, and PC3 for 11.8% (Table A4).

**FIGURE 6:**
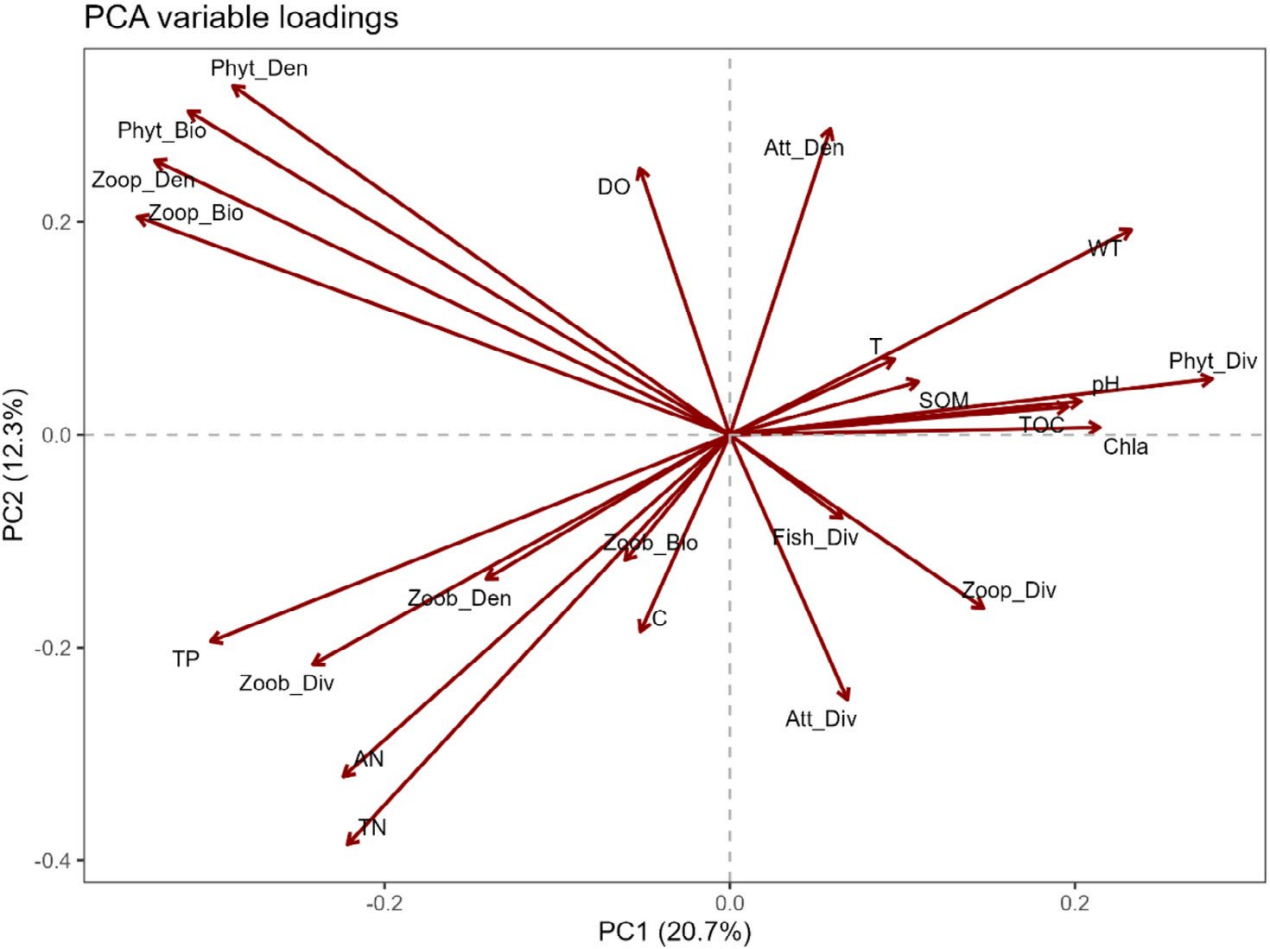
PCA variable loadings plot for the first two principal components (PC1 and PC2). Arrows represent the direction and contribution (loading) of each environmental and biotic variable. Percentages of variance explained are given in axis labels.

The PCA biplot (Figure 6) revealed gradients in environmental and biological conditions. PC1 was mainly associated with plankton‐related variables; Zoop_Den, Zoop_Bio, Phyt_Den, Phyt_Bio, and TP showed strong negative loadings, while Phyt_Div loaded positively. This axis represents a nutrient‐plankton productivity gradient, with higher PC1 scores indicating lower nutrient levels and plankton biomass. PC2 was driven by positive loadings of Phyt_Den, Phyt_Bio, Zoop_Den, Zoop_Bio, and DO, and negative loadings of TN and AN, reflecting a gradient in primary producer structure versus nitrogen nutrient availability. PC3 was characterized by positive loadings of Zoob_Den, Zoob_Bio, Chla, Zoop_Div, pH, and C, and negative loadings of Att_Div and Phyt_Div, capturing a water quality and trophic condition gradient associated with zooplankton density and zoobenthos density and photosynthetic activity. Overall, the PCA results indicate that variation in aquatic ecosystems was jointly structured by nutrient levels and plankton community dynamics.

#### 3.3.4 Responses of Guilds to PCA Gradients (Tweedie GLLMs)

All models passed diagnostic tests for overdispersion and zero‐inflation. Residual spatial autocorrelation was nonsignificant (Moran's I *p* > 0.05) except for M1 (Table 2). For guild_1 (Ecological guilds), the main‐effects model (M1) showed significant effects of PC1–PC3, but in the interaction model (M2) only PC1 remained significant and no interactions were detected. For guild_3 (IMEX), the main‐effects model (M5) again showed significant effects of PC1–PC3, while the interaction model (M6) retained only PC1 and PC2 with no significant interactions. Marginal *R* ^2^ for these schemes was consistently low (0.086–0.091). In contrast, the guild_2 (Feeding guild) scheme captured more structure. The interaction model (M4) revealed that piscivores had significantly lower baseline density than carnivores (*β* = −2.89, *p* = 0.028) and exhibited a markedly stronger positive response to PC2 (PC2 × PIS interaction: *β* = 0.673, *p* = 0.010). After accounting for species‐level heterogeneity, PC1 (*β* = 0.149, *p* = 0.003) and PC2 (*β* = 0.229, *p* = 0.001) remained significant predictors. The species random intercept alone explained a large share of the variance (*σ* ^2^ = 2.76, SD = 1.66), contributing to a conditional *R* ^2^ of 0.76 versus a marginal *R* ^2^ of 0.32, confirming that inter‐specific baseline differences are substantial yet do not obscure guild‐specific environmental responses.

**TABLE 2:** Comparison of six candidate GLMMs explaining guild density.

Model	Fixed effects structure	*R* ^2^_m_	*R* ^2^_c_	AIC	SME	SI	Res. Moran's I *p*
M1	PC1 + PC2 + PC3 + guild_1	0.086	0.69	1417.8	PC1**, PC2**, PC3*	\	0.046*
M2	(PC1 + PC2 + PC3) × guild_1	0.091	0.698	1420.6	PC1*	\	0.171
M3	PC1 + PC2 + PC3 + guild_2	0.171	0.696	1417.7	PC1**, PC2**, PC3*	\	0.145
M4	(PC1 + PC2 + PC3) × guild_2	0.318	0.763	1419.8	PC1**, PC2**, guild_2PIS*	PC2 × PIS, PIS*	0.231
M5	PC1 + PC2 + PC3 + guild_3	0.087	0.689	1417.8	PC1*, PC2*, PC3*	\	0.384
M6	(PC1 + PC2 + PC3) × guild_3	0.088	0.693	1422.3	PC1*, PC2**	\	0.136

*Note:* Significance codes: Significance codes: **p < 0.01; *p < 0.05. Model: Candidate model identifier (M1–M6). Fixed effects structure: Model formula specifying how fixed predictors (PC1–3, guild) are combined. PC1–3: First three principal components. guild: Categorical foraging guild variable. *R* ^2^_m_: Variance explained by fixed effects alone. *R* ^2^_c_: Variance explained by both fixed and random (species) effects. AIC: Akaike Information Criterion; lower values indicate better fit–complexity balance. SME: Significant Main Effects. Individual predictors with *p* < 0.05 or *p* < 0.01, ignoring interactions. SI: Significant Interactions. Terms indicating that the effect of one predictor depends on the level of another. Res. Moran's I *p*: Spatial autocorrelation test of model residuals; *p* < 0.05 indicates significant spatial structure remains, *p* ≥ 0.05 indicates spatial autocorrelation is adequately controlled. Diagnostic summary: All models passed DHARMa overdispersion and zero‐inflation tests (*p* > 0.05). M1 residuals exhibited significant spatial autocorrelation (*p* < 0.05).

Overall, environmental effects varied among classification schemes, and the CAR/PIS/OMN scheme (guild_2) best captured guild‐specific responses along PC2.

#### 3.3.5 Consistency and Divergence Among Models

The relationships between environmental variables and waterbird guild densities showed both similarities and differences across the three methods (RF, LASSO, PCA‐GLMM). Total nitrogen and SOM were negatively associated with multiple guilds (e.g., net‐exporter, waterfowl, and carnivorous) in both RF and LASSO (Table 3), and these patterns were partly reflected in the PCA‐GLMM results, where nutrient‐related principal components (e.g., PC2, driven by TN, AN and primary producer variables) showed significant associations with guild densities. In contrast, fish diversity and dissolved oxygen were positively associated with guild densities, particularly for piscivorous and carnivorous guilds. These variables likely represent relatively robust ecological linkages between waterbird guilds and environmental as well as biotic factors.

**TABLE 3:** Comparison of key environmental and aquatic predictors and their relationships with waterbird guilds based on Random Forest (RF) and LASSO models.

Independent variables	IMEX	EX	CAR	PIS	OMN	WF	W
AN		R−		R			
TP				R			−
TN	R	R−	−			R−	
Chla							−
TOC		+	+		R	R+	+
DO		+	R+			+	
WT	R		R+	R	R		R
pH							
T		+	+		R	+	R
C			+			+	
SOM		−	R−	R		R−	R−
Zoob_Den		+	+			+	
Zoob_Bio					R		
Zoob_Div	R	−				−	−
Fish_Div		+	R+	R	R	R+	R+
Zoop_Den		R+					
Zoop_Bio							
Zoop_Div			R+			+	
Phyt_Den							+
Phyt_Bio							
Phyt_Div		R					
Att_Den	R	R+	+			R	R+
Att_Div	R						

*Note:* Variables identified as important by random forest (RF) and their directional associations from LASSO regression across seven waterbird guilds. IMEX: Import‐exporter guild, EX: Net‐exporter guild, OMN: Omnivorous, PIS: Piscivorous, CAR: Carnivorous, W: Waders, WF: Waterfowl. Each cell summarizes results from both models. “R” = identified as important by RF; “+” = positive LASSO coefficient; “−” = negative LASSO coefficient; blank = not selected by either method. R alone indicates selection by RF only.

Compared with RF and LASSO, the PCA‐GLMM framework identified significant linear relationships between environmental gradients and bird density across all three guild classifications, although the strength and nature of these associations varied. For guild_2 (feeding guilds), the interaction model revealed that piscivorous birds responded more strongly to PC2 (i.e., higher algal biomass and dissolved oxygen, lower nitrogen) than other guilds, and this guild also exhibited a lower baseline density. The marginal *R* ^2^ of this model reached 0.318. Under guild_1 and guild_3, the main effects of PC1–PC3 were significant in the maineffects models, but interactions were not detected and the marginal *R* ^2^ values were modest. The inclusion of species‐specific random intercepts and the control of spatial autocorrelation in the GLMMs provide robust inference while accounting for data structure that RF and LASSO do not explicitly model. These methodological differences highlight complementary strengths: RF and LASSO are capable of identifying nonlinear, multivariable signals without strong distributional assumptions, but they may overfit, as reflected in the instability of LASSO for certain guilds (e.g., importer–exporter, omnivorous, and piscivorous). In contrast, PCA‐GLMM offers formal hypothesis testing and effect size estimation for predefined environmental gradients, at the cost of assuming additive or linear relationships and reduced power when sample size is limited (*n* = 33). The convergence of results across methods—particularly the importance of variables related to nutrient‐primary producer gradients and dissolved oxygen—underscores their ecological relevance for waterbird guilds.

## 4 Discussion

### 4.1 Key Environmental Associations With Waterbird Guild Patterns

Guilds have long been recognized as useful for understanding avian community structure and their responses to environmental associations (Root 1967; Simberloff and Dayan 1991). Previous studies showed that guild‐based analyses help explain how birds respond to environmental change (Canterbury et al. 2000; Chatterjee et al. 2020; Severinghaus 1981; Szaro 1986; Verner 1984). In this study, approaches as exploratory tools to examine potential associations between physicochemical properties, aquatic community structure metrics, and waterbird guilds patterns. RF and LASSO regression were applied to identify recurrent variables associated with variation in guild density, rather than to generate definitive predictions (Elith et al. 2008; Olden et al. 2008).

PCA‐GLMMs were used to test linear relationships between composite environmental gradients and guild density while accounting for species level heterogeneity. PC1 (20.7% of variance) represented a eutrophication gradient contrasting phytoplankton dominated conditions with zooplankton dominated conditions. PC2 (12.3%) separated sites with high algal biomass and dissolved oxygen from sites with elevated nitrogen (TN, AN) and low oxygen. PC3 (11.8%) primarily reflected zoobenthic productivity. PC1 and PC2 showed consistently significant positive main effects on waterbird density across all three guild classification schemes, indicating that guild level density tends to be higher in nutrient enriched, phytoplankton rich habitats with adequate dissolved oxygen.

The feeding guild (CAR/PIS/OMN) best captured guild specific responses: the interaction model revealed that piscivorous birds responded significantly more strongly to PC2 than carnivorous birds (PC2 × PIS: *β* = 0.673, *p* = 0.010), and this guild also showed a markedly lower baseline density (*β* = −2.892, *p* = 0.028). The marginal *R* ^2^ of this model (0.318) was substantially higher than those of the other classification schemes (0.086–0.091). This finding is strongly supported by the single variable pattern of fish diversity (Fish_Div), which was the biotic variable most consistently identified as important by RF and LASSO across multiple guilds (CAR, PIS, OMN, WF, W) and nearly always exhibited positive coefficients (Table 3). High values of PC2 correspond to high dissolved oxygen, which generally sustains richer fish communities (Mishra et al. 2023; Studds et al. 2012). Thus, the distinctive preference of piscivorous waterbirds for PC2 is consistent with a fish‐mediated trophic pathway (Dessborn et al. 2011; Elmberg et al. 2010).

The RF and LASSO results further corroborated these composite gradients at the individual variable level. The negative loading of TN on PC2 was matched by negative associations between TN and guild density across models, particularly for guilds using open‐water habitats such as EX and WF (Table 3). Elevated TN is commonly linked to eutrophication‐driven declines in water transparency and dissolved oxygen, which can reduce fish and invertebrate prey and thereby lower habitat suitability for waterbirds (Clark and Frid 2001; Rabalais 2002; Smith et al. 1999). Conversely, DO showed consistent positive associations with multiple guilds (EX, PIS, CAR, WF), likely acting through food availability rather than as a direct physiological factor. Therefore, the composite gradient represented by PC2, characterized by low nitrogen, high dissolved oxygen, and high primary productivity, constitutes the core environmental axis driving variation in waterbird guild density.

PC1 represents a gradient contrasting phytoplankton versus zooplankton dominance, and its positive effect indicates higher guild density in phytoplankton‐rich waters. This pattern is corroborated by the positive associations of attached algae density (Att_Den) with the EX, CAR, and W guilds in both RF and LASSO (Table 3). Although zooplankton density (Zoop_Den) showed a positive association with EX, the overall pattern aligns with the PC1 loading structure, suggesting that phytoplankton‐dominated waters may provide a more stable food base.

Water temperature (WT) was frequently selected by RF and LASSO, but its coefficient signs were unstable, suggesting that its effect may not be accurately characterized in this study (Ahola et al. 2004; Dunn and Winkler 1999), although warmer conditions can enhance invertebrate and fish growth in some contexts (Batzer and Wissinger 1996; Holopainen et al. 2015).

In contrast, PC3 primarily captured variation in zoobenthic productivity; although significant in most main‐effects models, its effect size was small and no significant interactions were detected. Similarly, RF and LASSO showed zoobenthos density (Zoob_Den) positively associated with only a few guilds (EX, CAR, WF), while zoobenthos diversity (Zoob_Div) was negatively associated or unrelated to several guilds. This suggests that zoobenthic resources exert a weaker overall constraint on waterbird guilds than fish and water redox conditions. Nevertheless, zoobenthos and periphyton have been shown to support benthic invertebrate communities and higher trophic levels in other systems (Cai et al. 2024; Jones et al. 1999). SOM was consistently associated with lower guild density in RF and LASSO (Aarif et al. 2025). Thus, the role of benthic pathways may be context‐dependent and warrants further investigation.

Overall, the key variables identified by RF and LASSO included TN, DO, SOM, Fish_Div, Att_Den. Their directional effects aligned clearly with the composite environmental gradients from PCA‐GLMM. This cross‐method consistency indicates that the integrated gradient of nutrients, productivity, and oxygen is the primary driver of spatial variation in waterbird guild density in Lake Nansi. This gradient operates mainly through changes in fish and invertebrate food resources.

### 4.2 Random Effects and Model Reliability

Accounting for species‐specific baseline densities was essential because gregarious species can otherwise dominate density sums and mask the responses of grouping or territorial species. After incorporating species as a random intercept into the model, we found that compositional differences among species accounted for the majority of the variation in density (Δ*R* ^2^≈0.44, *σ* ^2^ = 2.76). This quantitatively substantiates an important concern: if species densities are simply combined, high‐density gregarious species (e.g., *Chlidonias hybrida* ) would statistically overwhelm territorial species (e.g., various heron species), thereby obscuring the environmental responses of the latter (Korňan et al. 2019). Nonetheless, after controlling for species identity, PC1 and PC2 still exhibited significant main effects on density, and the effect of PC2 was stronger in the piscivorous guild, indicating that environmental gradients have detectable influences on all species, rather than simply reflecting shifts in species composition.

### 4.3 Model Consistency and Discrepancies

Despite their differing assumptions, the three methods identified broadly consistent associations between waterbird guild densities and environmental gradients. The composite gradient PC2, characterized by low nitrogen, high dissolved oxygen, and high primary productivity, emerged as the core environmental axis in PCA‐GLMM, and its constituent variables, TN (negative) and DO (positive), were among the most consistently selected predictors in RF and LASSO (Table 3). Similarly, fish diversity was the biotic variable most frequently identified as important across guilds by both RF and LASSO, which aligns with the stronger response of piscivorous birds to PC2 detected by the interaction GLMM. PC1, representing the phytoplankton versus zooplankton contrast, also showed consistent positive effects across models, corroborated by positive associations of attached algae density in RF and LASSO. Even the weaker role of PC3, reflecting zoobenthic productivity, was mirrored in the limited selection of variables related to zoobenthos by RF and LASSO.

Key environmental drivers were mostly consistent across models, although some models showed marked differences. LASSO performed poorly or was unstable for several guilds, such as IMEX, OMN, and PIS. These differences reflect both ecological mechanisms and methodological characteristics rather than model failure (John Lu 2010). On the one hand, the three methods differ in their assumptions about variable relationships. RF, based on ensemble decision trees, can automatically capture nonlinear relationships; PCA‐GLMM assumes linear or additive relationships between the response and predictors; LASSO also assumes linearity and selects variables through L1 regularization. When true ecological relationships are nonlinear, RF is more likely to detect them, whereas linear methods (LASSO, PCA‐GLMM) may miss or underestimate such signals. On the other hand, PCA‐GLMM controls for differences in baseline density among species through species‐specific random intercepts and evaluates spatial autocorrelation using Moran's I test, thereby accounting for spatial nonindependence in the data.

### 4.4 Ecological Consistency of Guild–Environment Relationships

The negative associations between nitrogen enrichment and waterbird guild density match results from other aquatic systems. Many studies show that high nutrient levels reduce waterbird numbers through habitat degradation caused by eutrophication (Laursen et al. 2025; Liu et al. 2025). The positive links between DO and several guilds also match earlier studies. These studies highlight the indirect role of oxygen in supporting aquatic prey (Mishra et al. 2023; Studds et al. 2012).

The weak and inconsistent influence of TP suggests that waterbird responses may reflect different eutrophication stages rather than simple nutrient gradients (Rönkä et al. 2005). Positive links between water transparency and piscivorous or carnivorous guilds also match past work. High turbidity often reduces feeding success for visual predators (Mallin et al. 2016; van Eerden et al. 1993).

Fish diversity again showed strong links with waterbird guild patterns. This result supports the use of waterbirds as indicators of fish communities and trophic status in lakes and wetlands (Dessborn et al. 2011; Elmberg et al. 2010). Together, these results support the use of guild‐based indicators to assess nutrient stress and food web condition in shallow lakes (Warren 2006).

### 4.5 Limitations and Future Directions

Species differ in diet and habitat use, so their responses to the same environmental drivers can vary in strength or direction. When grouped into guilds by these traits, guild‐level metrics such as density may show mixed or even opposing responses, weakening guild‐level signals. This problem can be amplified when sample sizes are small. For RF, the imbalance between samples and features increases overfitting risk: individual trees may memorize training data, and bagging cannot fully counteract this when samples are few, sometimes producing high training accuracy but poor test performance (e.g., Figure 4a,c,d,f). Random noise features may also be erroneously assigned high importance. For LASSO, the choice of *λ* is difficult and can shrink all coefficients to zero, preventing valid model construction for several guilds (IMEX, PIS, OMN) (Ranstam and Cook 2018). Moreover, LASSO tends to arbitrarily select one variable from a correlated group, making the set of selected features unstable across repeated runs. For PCA, small samples may cause noise directions to be misidentified as principal components, diluting true signals. For GLMMs, the limited sample size reduces the reliability of variance estimates and lowers statistical power for detecting interactions; the significant PC2 × PIS interaction (*p* = 0.010) is therefore noteworthy, but the absence of interactions for other guild schemes cannot be taken as evidence of their nonexistence. In summary, while each model has inherent limitations under small sample conditions, the convergence of key findings across methods, particularly the consistent importance of the nutrient, productivity and oxygen gradient, suggests that these limitations do not undermine the main ecological conclusions of this study.

This study is limited by its single year, single season design and the modest sample size, which restrict inference on seasonal dynamics and reduce statistical power. Future work should employ multi‐year and multi‐season sampling to capture the temporal variability of both environmental conditions and waterbird communities. Increasing the number of sampling sites, preferably along a wider trophic gradient, would also strengthen statistical power. Additionally, incorporating trait‐based or phylogenetic approaches alongside guild classification may offer a more mechanistic understanding of species' environment relationships.

### 4.6 Implications for Wetland Management

This study consistently identified nutrients and aquatic biota as key correlates of waterbird guild patterns. This result shows the value of guild‐based indicators for wetland assessment. Guild metrics combine responses from many species. These metrics can give clear signals of trophic status and ecosystem condition. Managers can include guild‐level indicators in monitoring programs (Carignan and Villard 2002). This approach can help detect nutrient stress early. It can also support ecosystem‐based management in shallow lakes such as Nansi Lake.

## 5 Conclusions

This study provides the first guild‐level assessment of waterbird environment relationships in Nansi Lake and identifies total nitrogen, SOM, periphyton, and fish diversity as key correlates of guild density. Accounting for baseline density differences among species was essential for detecting these environmental gradients, as gregarious species would otherwise mask the responses of territorial taxa.

## Author Contributions

**Sai Jiang:** conceptualization (equal), data curation (equal), formal analysis (equal). **Yuewei Yang:** methodology (equal), project administration (equal). **Fengfei Sun:** formal analysis (equal), funding acquisition (equal), validation (equal), writing – original draft (equal). **Xingjia Zheng:** funding acquisition (equal), supervision (equal), validation (equal). **Wei He:** conceptualization (equal), formal analysis (equal), resources (equal). **Meizhen Tang:** data curation (equal), formal analysis (equal), writing – original draft (equal). **Junfeng Chen:** formal analysis (equal), funding acquisition (equal), supervision (equal), validation (equal), writing – review and editing (equal). **Shiqiang Ma:** funding acquisition (equal), visualization (equal). **Gang Li:** investigation (equal), software (equal), supervision (equal).

## Funding

This work was supported by the National Natural Science Foundation of China, NO. 31672314.

## Conflicts of Interest

The authors declare no conflicts of interest.

## Supporting information


**Table A1** Environmental variables, aquatic biotic indices, and waterbird guild densities (log(*x* + 1)‐transformed) at 33 sampling sites in Nansi Lake.
**Table A2**. Complete species list of observed waterbirds (Nspecies = 17) with their guild classifications. Guilds: CAR, Carnivorous; EX, Net‐exporter; IMEX, Importer‐exporter; OMN, Omnivorous; PIS, Piscivorous; W, Wader; WF, Waterfowl. Species names flollow Gill et al. (2021).
**Table A3**. Pairwise comparisons of waterbird guild densities using Wilcoxon rank‐sum tests.
**Table A4**. Loadings of environmental and biological variables on the first three principal components (PC1–PC3).
**Figure S1:** OOB error curves for the five validated RF model guilds. (a) Import‐exporter guild, (b) Net‐exporter guild, (c) Omnivorous, (d) Piscivorous, (e) Carnivorous, (f) Waders, (g) Waterfowl. Optimal trees: Minimum tree counted for Out‐of‐Bag (OOB) error stabilization.
**Figure S2:** CV error curve for LASSO regression. Optimal *λ* marked by red dashed line. (a) Import‐exporter guild, (b) Net‐exporter guild, (c) Omnivorous, (d) Piscivorous, (e) Carnivorous, (f) Waders, (g) Waterfowl.
**Figure S3:** Coefficient paths for each LASSO model. The red dashed line indicates the optimal lambda value. (a) Import‐exporter guild, (b) Net‐exporter guild, (c) Omnivorous, (d) Piscivorous, (e) Carnivorous, (f) Waders, (g) Waterfowl.
**Figure S4:** Correlation between aquatic biological community indicators and water quality parameters. Significance was indicated as follows: *p ≤ 0.05; **p ≤ 0.01.
**Figure S5:** Variable importance ranking based on %IncMSE from RF models for: (a) Import‐exporter guild, (b) Net‐exporter guild, (c) Omnivorous, (d) Piscivorous, (e) Carnivorous, (f) Waders, (g) Waterfowl.

## Data Availability

All the necessary code and data to reproduce this paper are provided as Supporting Information for the review process.
