# Supplementary material for: A Guild‐Based Ecological Assessment: Integrating Multi‐Model Approaches to Reveal Key Environmental Associations for Waterbirds in Nansi Lake, China
Source: Ecol Evol. 2026 May 21;16(5):e73694. doi: 10.1002/ece3.73694 (PMC13239128; doi:10.1002/ece3.73694)
Supplement: Supplementary file 1 — Table A1 Environmental variables, aquatic biotic indices, and waterbird guild densities (log(x + 1)‐transformed) at 33 sampling sites in Nansi Lake. Table A2. Complete species list of observed waterbirds (Nspecies = 17) with their guild classifications. Guilds: CAR, Carnivorous; EX, Net‐exporter; IMEX, Importer‐exporter; OMN, Omnivorous; PIS, Piscivorous; W, Wader; WF, Waterfowl. Species names flollow Gill et al. (2021). Table A3. Pairwise comparisons of waterbird guild densities using Wilcoxon rank‐sum tests. Table A4. Loadings of environmental and biological variables on the first three principal components (PC1–PC3). Figure S1: OOB error curves for the five validated RF model guilds. (a) Import‐exporter guild, (b) Net‐exporter guild, (c) Omnivorous, (d) Piscivorous, (e) Carnivorous, (f) Waders, (g) Waterfowl. Optimal trees: Minimum tree counted for Out‐of‐Bag (OOB) error stabilization. Figure S2: CV error curve for LASSO regression. Optimal λ marked by red dashed line. (a) Import‐exporter guild, (b) Net‐exporter guild, (c) Omnivorous, (d) Piscivorous, (e) Carnivorous, (f) Waders, (g) Waterfowl. Figure S3: Coefficient paths for each LASSO model. The red dashed line indicates the optimal lambda value. (a) Import‐exporter guild, (b) Net‐exporter guild, (c) Omnivorous, (d) Piscivorous, (e) Carnivorous, (f) Waders, (g) Waterfowl. Figure S4: Correlation between aquatic biological community indicators and water quality parameters. Significance was indicated as follows: *p ≤ 0.05; **p ≤ 0.01. Figure S5: Variable importance ranking based on %IncMSE from RF models for: (a) Import‐exporter guild, (b) Net‐exporter guild, (c) Omnivorous, (d) Piscivorous, (e) Carnivorous, (f) Waders, (g) Waterfowl. [file ECE3-16-e73694-s001.zip › ece373694-sup-0001-TableA1.pdf]

Table A1. Environmental variables, aquatic biotic indices, and waterbird guild densities (log(x+1)-transformed) at 33 sampling sites in Nansi Lake.

AN	TP	TN	Chla	TOC	DO	WT	pH	T	C	O	Zoob_Den	Zoob_Bio
0.982	0.199	1.601	2.944	1.841	1.917	3.223	2.140	3.091	6.944	3.205	6.344	1.454
0.867	0.223	1.664	2.639	1.649	1.841	3.235	2.140	2.944	7.286	4.501	7.474	5.352
0.908	0.293	1.366	3.178	1.649	1.887	3.336	2.140	2.197	7.001	1.716	7.388	6.946
0.360	0.255	1.423	2.485	1.649	1.974	3.231	2.140	3.178	6.306	2.600	7.633	5.488
0.779	0.239	1.345	3.466	1.668	2.001	3.307	2.140	3.497	6.792	4.755	8.774	6.433
0.713	0.215	1.163	3.091	1.649	1.960	3.339	2.140	3.989	6.999	4.061	7.867	3.923
0.574	0.113	1.212	3.135	1.649	1.988	3.311	2.163	4.159	6.690	3.222	6.799	1.439
1.203	0.322	1.509	3.296	1.872	1.946	3.288	2.140	4.078	7.186	4.236	8.794	4.668
0.693	0.104	1.278	3.497	1.668	1.917	3.321	2.128	4.007	6.810	3.293	5.932	2.869
0.959	0.262	1.200	2.398	1.668	1.856	3.325	2.128	3.807	7.157	2.939	4.796	0.190
0.642	0.140	1.051	3.258	1.808	1.841	3.325	2.163	4.174	7.098	2.650	2.197	0.391
0.723	0.157	1.495	4.025	1.841	2.054	3.364	2.163	3.761	6.783	4.598	7.408	6.144
0.820	0.223	1.179	3.367	1.740	1.841	3.339	2.152	3.784	7.074	4.653	7.029	4.687
0.779	0.174	1.154	3.761	1.775	1.974	3.346	2.152	3.555	6.948	3.825	5.932	4.259
0.779	0.215	1.125	3.178	1.740	2.028	3.336	2.140	3.219	6.720	5.368	6.790	5.343
1.082	0.270	1.351	3.296	1.932	1.917	3.311	2.175	3.912	6.718	3.959	4.394	3.106
0.589	0.307	1.520	3.714	1.609	1.932	3.329	2.104	3.367	6.172	4.062	6.613	4.178
0.928	0.270	1.099	3.401	2.028	1.808	3.300	2.140	4.277	6.672	3.729	6.187	5.098
0.884	0.174	1.366	3.332	1.887	1.974	3.318	2.128	3.555	6.506	4.610	5.889	3.390
0.583	0.086	1.163	4.718	2.001	1.974	3.311	2.197	4.094	6.898	4.747	8.435	6.265
0.565	0.140	1.054	3.989	1.705	1.775	3.314	2.175	4.290	6.823	3.866	5.517	4.913
0.441	0.077	1.044	3.892	1.758	1.974	3.343	2.175	4.007	6.692	4.367	5.836	4.299
0.591	0.068	1.666	1.946	1.740	1.723	3.325	2.152	4.234	6.870	4.152	5.609	2.422
0.620	0.157	0.963	2.079	1.841	1.856	3.281	2.116	0.000	6.871	4.407	5.380	3.849
0.669	0.174	1.454	2.708	1.609	1.917	3.311	2.128	3.784	6.612	4.644	4.679	2.322
0.422	0.095	0.952	5.050	2.079	1.825	3.339	2.241	3.367	6.755	4.534	5.549	3.675
0.416	0.148	0.908	1.792	1.792	1.856	3.325	2.152	4.174	6.927	3.687	4.977	2.896
0.513	0.140	1.135	2.944	1.668	1.932	3.325	2.128	3.219	6.715	4.526	4.654	2.927
0.265	0.095	0.978	2.079	1.740	2.001	3.346	2.128	4.304	6.737	4.799	5.720	5.505
0.353	0.086	1.511	2.303	1.504	1.974	3.325	2.128	3.892	6.509	2.805	5.746	4.446
0.272	0.058	0.531	2.565	2.015	2.028	3.353	2.116	3.219	6.830	3.858	5.911	3.940
0.728	0.223	1.144	2.639	1.841	1.974	3.346	2.152	3.401	6.764	1.898	6.142	5.581
0.372	0.068	0.916	2.485	1.887	2.028	3.325	2.140	3.850	6.929	4.909	5.056	1.610

Zoob_Div	Fish_Div	Zoop_Den	Zoop_Bio	Zoop_Div	Phyt_Den	Phyt_Bio	Phyt_Div	Att_Den	Att_Div	IMEX	EX	CAR
1.273	1.241	3.593	0.185	1.516	6.385	2.718	1.658	3.781	1.611	1.466	2.872	2.737
1.425	1.166	5.551	0.973	1.322	6.130	1.925	1.659	4.965	1.512	1.170	2.291	2.398
0.882	0.000	3.886	0.541	1.429	7.190	2.734	1.672	4.686	1.592	2.663	2.398	2.398
1.181	1.195	6.529	1.617	0.832	7.944	3.640	1.711	6.782	1.312	2.172	2.872	3.096
1.294	1.256	3.296	0.090	1.340	7.328	3.217	1.681	4.915	1.373	0.000	3.145	2.933
1.147	1.228	3.729	0.219	1.452	7.004	2.646	1.629	5.662	0.986	0.000	2.097	0.000
0.689	1.278	5.737	1.099	1.501	7.867	3.149	1.663	5.268	1.571	2.097	4.421	4.421
1.210	1.138	5.395	0.819	1.427	7.497	3.116	1.668	6.390	1.436	0.000	4.331	4.331
1.166	1.004	5.527	0.953	1.385	8.124	3.011	1.626	5.080	1.359	2.933	4.129	4.338
0.000	1.252	3.850	0.230	1.357	7.202	2.867	1.607	7.137	1.437	3.397	4.074	4.231
1.166	1.307	5.514	0.890	1.427	7.368	2.830	1.664	5.295	1.407	2.097	4.542	4.615
1.022	1.315	4.312	0.363	1.511	6.875	2.289	1.658	5.295	1.571	3.387	3.110	3.859
0.893	1.455	5.319	0.844	1.592	7.679	2.997	1.702	5.528	1.485	3.320	3.887	4.263
1.195	1.244	5.648	1.086	1.419	8.029	3.376	1.664	5.662	1.332	2.097	3.110	3.387
0.732	0.000	5.416	0.693	1.460	7.931	3.669	1.763	5.528	1.512	0.000	2.461	2.461
1.046	1.376	5.561	1.109	1.428	7.221	3.147	1.744	4.686	1.594	0.000	2.663	2.582
1.089	1.433	5.413	0.974	1.364	5.657	1.487	1.715	2.484	1.706	0.000	3.696	3.258
1.243	1.344	5.441	0.896	1.008	5.884	2.250	1.736	5.295	1.594	2.172	3.320	3.320
1.158	1.376	5.209	0.759	1.181	5.679	2.039	1.733	5.528	1.593	0.000	3.360	3.145
0.514	1.324	2.054	0.038	1.525	5.696	1.732	1.788	5.430	1.451	2.097	5.467	5.497
1.025	1.247	3.818	0.292	1.371	5.558	1.770	1.740	5.752	1.282	0.000	3.110	3.110
0.797	1.154	2.366	0.036	1.503	5.620	1.605	1.793	5.752	1.593	1.880	3.434	3.397
1.258	1.321	4.091	0.345	1.401	5.134	1.282	1.773	5.125	1.693	0.000	2.461	2.461
0.936	1.048	2.485	0.044	1.507	5.314	1.627	1.779	5.771	1.593	2.037	2.582	2.737
1.008	1.268	2.833	0.077	1.415	5.383	1.366	1.804	5.771	1.718	0.000	2.172	2.037
0.336	1.305	2.497	0.055	1.405	5.840	1.687	1.756	6.401	1.407	2.663	3.629	3.932
0.602	1.130	2.639	0.062	1.383	6.446	2.045	1.738	7.097	1.407	3.859	4.503	4.716
1.007	1.244	3.170	0.111	1.288	6.883	2.675	1.708	6.938	1.332	3.360	3.974	3.995
0.985	1.145	5.085	0.638	1.442	7.371	3.019	1.785	6.401	1.593	3.469	5.217	4.909
1.067	0.832	3.982	0.283	1.284	5.620	1.723	1.733	5.640	1.593	0.000	3.110	2.937
0.607	1.218	4.596	0.336	1.288	6.444	2.242	1.769	6.401	1.407	5.150	5.191	5.707
1.211	1.115	4.706	0.485	1.323	6.047	1.907	1.765	6.401	1.593	6.416	5.633	6.717
1.286	1.048	5.434	0.627	1.201	6.638	2.397	1.738	6.401	1.613	0.000	6.905	6.905

OMN	PIS	WF	W
1.880	0.000	2.872	1.466
0.000	0.000	1.695	2.037
1.466	0.000	2.663	2.398
0.747	1.170	2.582	2.582
1.695	0.000	2.872	1.880
0.000	2.097	2.097	0.000
0.000	2.097	4.377	2.461
0.000	0.000	4.232	2.097
0.000	1.466	3.974	3.320
2.990	0.000	4.181	3.145
0.000	0.000	4.542	2.097
1.520	0.000	3.110	3.387
0.747	1.170	3.792	3.469
0.000	0.000	3.110	2.097
0.000	0.000	2.461	0.000
0.000	0.747	2.663	0.000
2.727	0.000	3.696	0.000
1.880	0.747	2.872	2.933
1.170	1.466	3.279	1.170
0.000	0.000	5.467	2.097
0.000	0.000	3.110	0.000
1.880	0.747	3.360	2.172
0.000	0.000	1.520	2.097
1.170	0.000	2.398	2.291
0.747	0.000	1.695	1.466
0.000	0.000	3.469	2.990
1.520	3.110	4.377	4.063
3.320	0.000	4.147	2.933
3.599	3.792	5.161	3.740
0.000	1.520	2.727	2.097
2.097	3.696	5.170	5.170
3.932	2.727	5.581	6.439
0.000	0.000	6.905	0.000
