# Supplementary material for: A Guild‐Based Ecological Assessment: Integrating Multi‐Model Approaches to Reveal Key Environmental Associations for Waterbirds in Nansi Lake, China
Source: Ecol Evol. 2026 May 21;16(5):e73694. doi: 10.1002/ece3.73694 (PMC13239128; doi:10.1002/ece3.73694)
Supplement: Supplementary file 1 — Table A1 Environmental variables, aquatic biotic indices, and waterbird guild densities (log(x + 1)‐transformed) at 33 sampling sites in Nansi Lake. Table A2. Complete species list of observed waterbirds (Nspecies = 17) with their guild classifications. Guilds: CAR, Carnivorous; EX, Net‐exporter; IMEX, Importer‐exporter; OMN, Omnivorous; PIS, Piscivorous; W, Wader; WF, Waterfowl. Species names flollow Gill et al. (2021). Table A3. Pairwise comparisons of waterbird guild densities using Wilcoxon rank‐sum tests. Table A4. Loadings of environmental and biological variables on the first three principal components (PC1–PC3). Figure S1: OOB error curves for the five validated RF model guilds. (a) Import‐exporter guild, (b) Net‐exporter guild, (c) Omnivorous, (d) Piscivorous, (e) Carnivorous, (f) Waders, (g) Waterfowl. Optimal trees: Minimum tree counted for Out‐of‐Bag (OOB) error stabilization. Figure S2: CV error curve for LASSO regression. Optimal λ marked by red dashed line. (a) Import‐exporter guild, (b) Net‐exporter guild, (c) Omnivorous, (d) Piscivorous, (e) Carnivorous, (f) Waders, (g) Waterfowl. Figure S3: Coefficient paths for each LASSO model. The red dashed line indicates the optimal lambda value. (a) Import‐exporter guild, (b) Net‐exporter guild, (c) Omnivorous, (d) Piscivorous, (e) Carnivorous, (f) Waders, (g) Waterfowl. Figure S4: Correlation between aquatic biological community indicators and water quality parameters. Significance was indicated as follows: *p ≤ 0.05; **p ≤ 0.01. Figure S5: Variable importance ranking based on %IncMSE from RF models for: (a) Import‐exporter guild, (b) Net‐exporter guild, (c) Omnivorous, (d) Piscivorous, (e) Carnivorous, (f) Waders, (g) Waterfowl. [file ECE3-16-e73694-s001.zip › ece373694-sup-0002-supinfo.pdf]

Table A2. Complete species list of observed waterbirds ($N_{species}$=17) with their guild classifications. Guilds: IMEX, Importer-exporter; EX, Net-exporter; CAR, Carnivorous; OMN, Omnivorous; PIS, Piscivorous; W, Wader; WF, Waterfowl. Species names flollow Gill et al., 2021.

Scientific Name	Order	Authority	Guild1	Guild2	Guild3
Chlidonias leucopterus	Charadriiformes	(Temminck, 1815)	EX	CAR	WF
Chlidonias hybrida	Charadriiformes	(Pallas, 1811)	EX	CAR	WF
Ardea cinerea	Pelecaniformes	Linnaeus, 1758	IMEX	CAR	W
Ardea purpurea	Pelecaniformes	Linnaeus, 1766	IMEX	CAR	W
Ardeola bacchus	Pelecaniformes	(Bonaparte, 1855)	EX	CAR	W
Ardea alba	Pelecaniformes	Linnaeus, 1758	IMEX	CAR	W
Ixobrychus sinensis	Pelecaniformes	Gmelin, JF, 1789	EX	CAR	W
Bubulcus coromandus	Pelecaniformes	(Boddaert, 1783)	IMEX	CAR	W
Nycticorax nycticorax	Pelecaniformes	(Linnaeus, 1758)	EX	CAR	W
Ardea intermedia	Pelecaniformes	Wagler, 1829	IMEX	CAR	W
Egretta garzetta	Pelecaniformes	(Linnaeus, 1766)	IMEX	CAR	W
Gallinula chloropus	Gruiformes	(Linnaeus, 1758)	EX	OMN	WF
Fulica atra	Gruiformes	Linnaeus, 1758	EX	OMN	WF
Phalacrocorax carbo	Suliformes	(Linnaeus, 1758)	EX	PIS	WF
Anas zonorhyncha	Anseriformes	Swinhoe, 1866	IMEX	OMN	WF
Podiceps cristatus	Podicipediformes	(Linnaeus, 1758)	EX	PIS	WF
Tachybaptus ruficollis	Podicipediformes	(Pallas, 1764)	EX	PIS	WF

References

Boros E (2021): Classification method for quantification of waterbird nutrient cycling guilds. MethodsX (8): 101597. https://doi.org/10.1016/j.mex.2021.101597

Gill, F., Donsker, D., Rasmussen, P., (Eds). 2021. IOC World Bird Li st (v 11.2). Doi 10.14344/IOC.ML.11.2. http://www.worldbirdnames.org/

Table A3. Pairwise comparisons of waterbird guild densities using Wilcoxon rank-sum tests.

	Guilds (density)	Z-value	p-value (Unadjusted)
Guild1: comparison results of nutrient cycling and transport guilds	EX_Den - IMEX_Den	-4.351	p<0.001
	OMN_Den - CAR_Den	-4.937	p<0.001
Guild2: comparison results of feeding guilds	PIS_Den - CAR_Den	-4.976	p<0.001
	PIS_Den - OMN_Den	-0.609	0.542
Guild3: comparison results of ecological guilds	W_Den - WF_Den	-3.92	p<0.001

Note: Densities were log(x+1)-transformed prior to analysis.

Table A4. Loadings of environmental and biological variables on the first three principal components (PC1-PC3).

**Variable**	**PC1**	**PC2**	**PC3**
TP	-0.301		
TN	-0.222	-0.385	
AN	-0.224	-0.321	0.224
Chla	0.214		0.316
TOC	0.196		
WT	0.233	0.193	
pH	0.204		0.304
DO		0.251	
Zoop_Den	-0.333	0.258	
Zoop_Bio	-0.343	0.205	
Phyt_Den	-0.288	0.328	0.213
Phyt_Bio	-0.314	0.304	0.203
Phyt_Div	0.279		-0.220
Zoob_Den			0.354
Zoob_Bio			0.262
Zoop_Div			0.307
C			0.317
Att_Div		-0.249	-0.308

Note: Only loadings with absolute values ≥ 0.20 are shown. Positive and negative values indicate the direction of the relationship between variables and principal components.

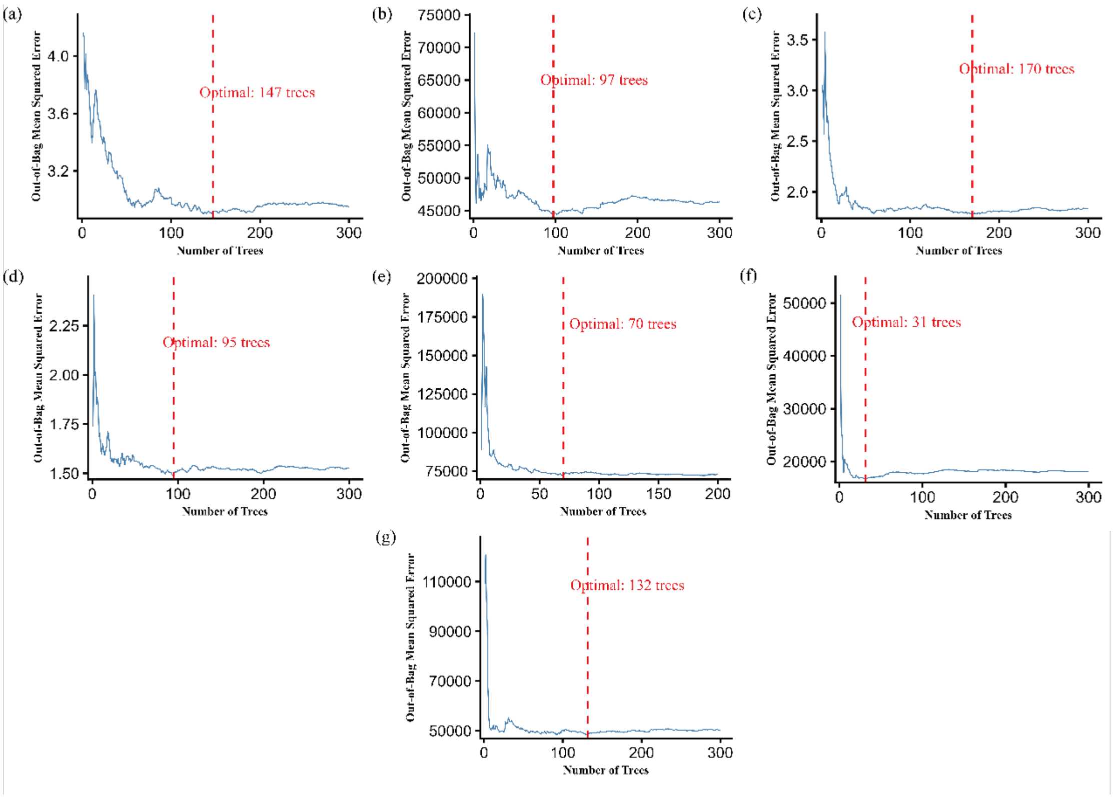

Figure S1. OOB error curves for the five validated RF model guilds. (a) Import-exporter guild, (b) Net-exporter guild, (c) Omnivorous, (d) Piscivorous, (e) Carnivorous, (f) Waders, (g) Waterfowl. Optimal trees: Minimum tree counted for Out-of-Bag (OOB) error stabilization.

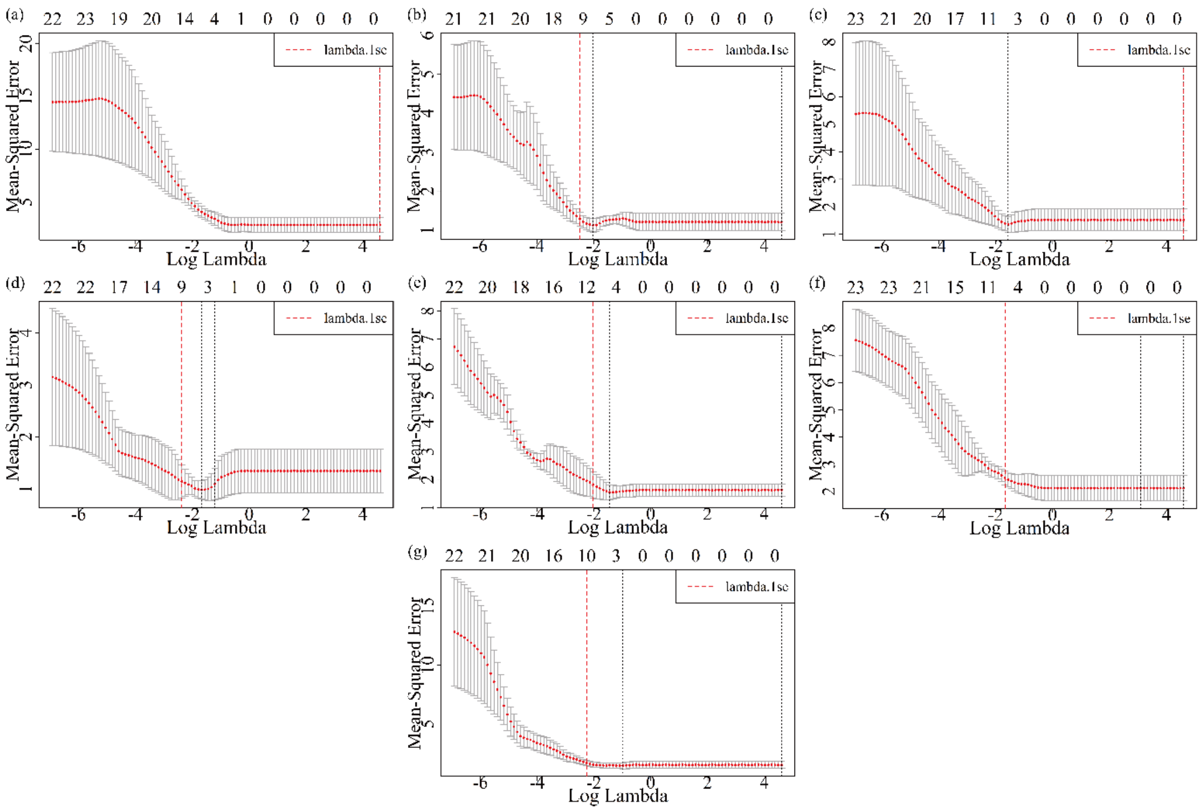

Figure S2. CV error curve for LASSO regression. Optimal λ marked by red dashed line. (a) Import-exporter guild, (b) Net-exporter guild, (c) Omnivorous, (d) Piscivorous, (e) Carnivorous, (f) Waders, (g) Waterfowl.

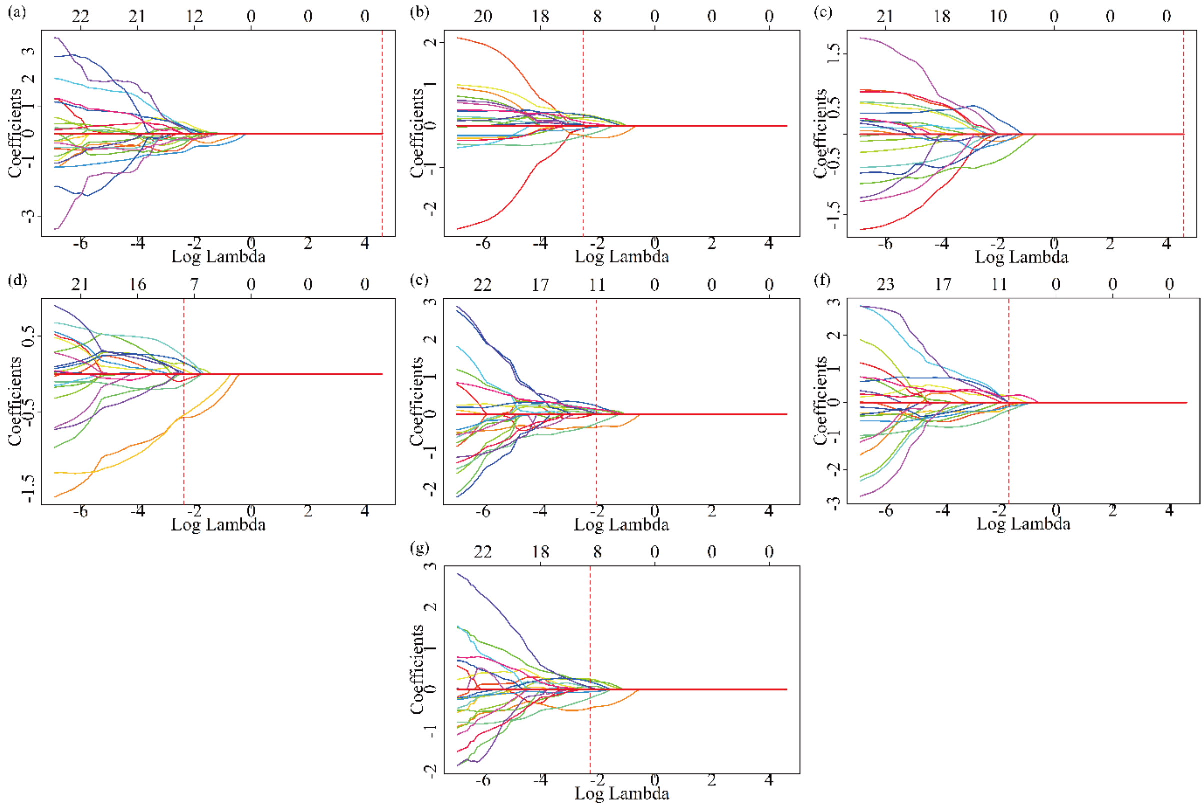

Figure S3. Coefficient paths for each LASSO model. The red dashed line indicates the optimal lambda value. (a) Import-exporter guild, (b) Net-exporter guild, (c) Omnivorous, (d) Piscivorous, (e) Carnivorous, (f) Waders, (g) Waterfowl.

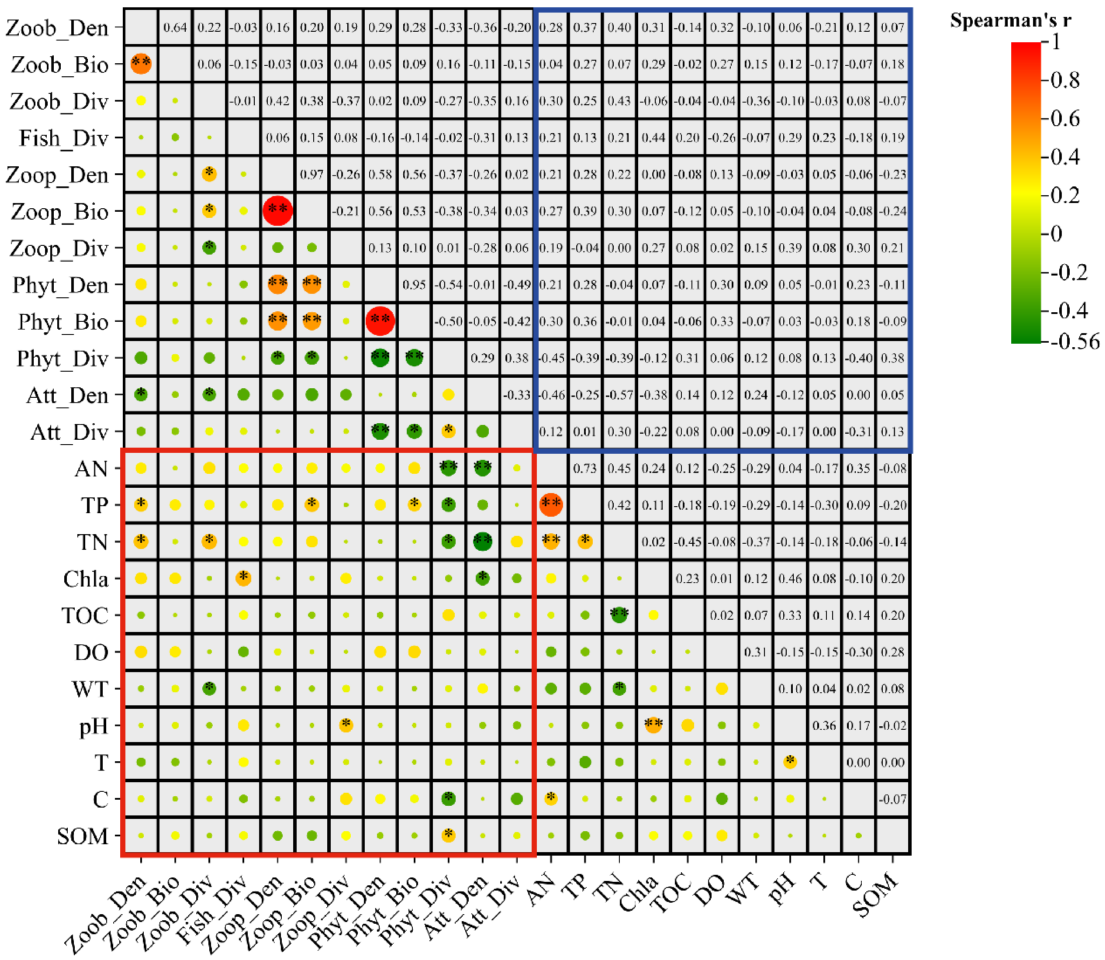

Figure S4. Correlation between aquatic biological community indicators and water quality parameters. Significance was indicated as follows: *$P \leq 0.05$; **$P \leq 0.01$.

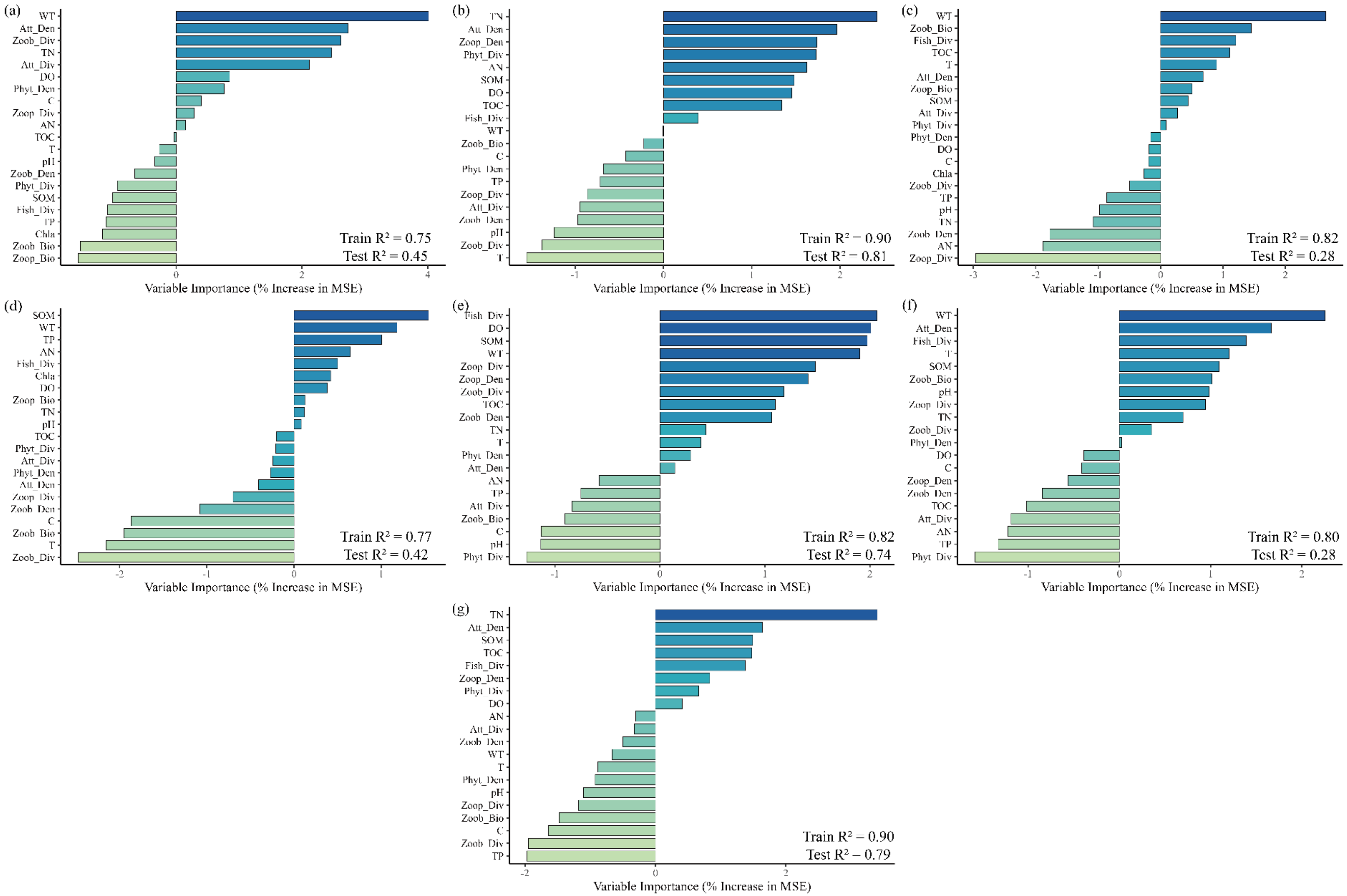

Figure S5. Variable importance ranking based on %IncMSE from RF models for: (a) Import-exporter guild, (b) Net-exporter guild, (c) Omnivorous, (d) Piscivorous, (e) Carnivorous, (f) Waders, (g) Waterfowl.
